# Case management and self-management support for frequent users with chronic disease in primary care: a pragmatic randomized controlled trial

**DOI:** 10.1186/1472-6963-13-49

**Published:** 2013-02-07

**Authors:** Maud-Christine Chouinard, Catherine Hudon, Marie-France Dubois, Pasquale Roberge, Christine Loignon, Éric Tchouaket, Martin Fortin, Éva-Marjorie Couture, Maxime Sasseville

**Affiliations:** 1 Département des sciences de la santé, Université du Québec à Chicoutimi; 555, boul. de l’Université, Chicoutimi, Québec G7H 2B1, Canada; 2 Centre de santé et de services sociaux de Chicoutimi, Québec, Canada; 3 Département de médecine de famille et de médecine d’urgence, Université de Sherbrooke, Québec, Canada; 4 Département des sciences de la santé communautaire, Université de Sherbrooke, Québec, Canada; 5 Département des sciences infirmières, Université du Québec en Outaouais, Québec, Canada; 6 Université de Sherbrooke, Québec, Canada

**Keywords:** Chronic diseases, Primary care, Family Medicine Group, Frequent users, Case management, Self-management, Primary care nursing, Services integration

## Abstract

**Background:**

Chronic diseases represent a major challenge for health care and social services. A number of people with chronic diseases require more services due to characteristics that increase their vulnerability. Given the burden of increasingly vulnerable patients on primary care, a pragmatic intervention in four Family Medicine Groups (primary care practices in Quebec, Canada) has been proposed for individuals with chronic diseases (diabetes, cardiovascular diseases, respiratory diseases, musculoskeletal diseases and/or chronic pain) who are frequent users of hospital services. The intervention combines case management by a nurse with group support meetings encouraging self-management based on the Stanford Chronic Disease Self-Management Program. The goals of this study are to: (1) analyze the implementation of the intervention in the participating practices in order to determine how the various contexts have influenced the implementation and the observed effects; (2) evaluate the proximal (self-efficacy, self-management, health habits, activation and psychological distress) and intermediate (empowerment, quality of life and health care use) effects of the intervention on patients; (3) conduct an economic analysis of the efficiency and cost-effectiveness of the intervention.

**Methods/Design:**

The analysis of the implementation will be conducted using realistic evaluation and participatory approaches within four categories of stakeholders (Family Medicine Group and health centre management, Family Medicine Group practitioners, patients and their families, health centre or community partners). The data will be obtained through individual and group interviews, project documentation reviews and by documenting the intervention. Evaluation of the effects on patients will be based on a pragmatic randomized before-after experimental design with a delayed intervention control group (six months). Economic analysis will include cost-effectiveness and cost-benefit analysis.

**Discussion:**

The integration of a case management intervention delivered by nurses and self-management group support into primary care practices has the potential to positively impact patient empowerment and quality of life and hopefully reduce the burden on health care. Decision-makers, managers and health care professionals will be aware of the factors to consider in promoting the implementation of this intervention into other primary care practices in the region and elsewhere.

**Trial Registration:**

NCT01719991

## Background

Because of their high prevalence and significant effects, chronic diseases (CD) pose a major challenge for health care and social services, for society and for the persons affected [1]. These individuals often have to make important day-to-day adjustments as a result of disability, loss of income, and a declining quality of life [2,3]. It is now recognized that primary care should be central to the customized and effective management of CD [4,5], and that innovative strategies targeting the current organization of primary care must be offered and evaluated to better support the individuals affected [1], particularly the most vulnerable [6]. To meet the complex needs of people with CD and to reduce societal consequences, primary care must provide a range of services that are interdisciplinary, person-centred and adjusted to the individual’s current health conditions and characteristics [7,8]. These services must also be oriented towards self-management, where the people affected and their families are called upon to play a greater role in the management of their health [1,7,9].

A number people with CD require higher intensity care because of personal characteristics that increase their vulnerability. This applies especially to the socioeconomically disadvantaged [10,11] and to those who present a comorbid mental health condition [12,13] or multimorbidity (two or more CD) [13,14]. In addition to a compromised quality of life and an increased risk of social isolation, these individuals have problems complying with treatment, adopting healthy behaviours and managing their health. This can result in increased services use, like emergency department visits and hospitalizations [15,16]. Case management strategies could be developed to address the vulnerability factors of frequent users in order to prevent inequities in health care and related costs [17].

In 2004, Family Medicine Groups (FMG) were implemented by the *ministère de la santé et des services sociaux du Québec* (Quebec’s ministry of health and social services) to improve accessibility, continuity, and coordination of health care in Quebec [18]. A FMG is an administrative arrangement for existing practices in which primary care physicians who wish to participate are grouped together to collaborate with nurses to offer primary care services, including patient follow-up, health promotion and preventive care, to a group of registered patients. It offers access to care 10 hours a day, seven days a week, through regular appointments, walk-in clinics, home visits, and after-hours health coverage using telephone hot-lines and emergency on-call services. Family physicians who are members of FMG will also work closely with other health care professionals in community services centres, hospitals, community pharmacies, etc., to complement the services they offer [19]. Since the creation of these new care teams, FMG nurses have already improved health education and the accessibility and continuity of services for certain patients, including those with diabetes, hypertension, on anticoagulant treatment, etc. [20]. However, the most vulnerable groups still pose major challenges in terms of accessibility, delivery and coordination of primary care [6]. The current work organization in FMG cannot optimally respond to the multiple requests, the considerable needs for self-management support of these patient groups, and their frequent need to access various health care resources [21], due, among other things, to lack of coordination and integration of services.

A major consultation process conducted in 2010 on the organization of CD services in the Saguenay-Lac-Saint-Jean (SLSJ) region of Québec, identified two potential solutions to meet the challenges posed by vulnerable patients with CD who frequently use hospital services [22]: (1) Improve the coordination of services through case management; and (2) Develop strategies to support self-management. Some vulnerable patients could benefit from closer monitoring by a case management nurse [1,23] within a primary care team linked to other network resources [1,6], and from self-management support [2,6]. Case management programs for frequent users of emergency departments have already been developed or are being developed in the six *Centre de santé et de services sociaux* (CSSS) (health and social services centres) of the SLSJ region, including the two CSSS participating in the project. Without being formally evaluated, an assessment of their programs has brought to light several positive points [24]: a significant decrease in the number of frequent users of emergency services; a high level of satisfaction among users and stakeholders; and close cooperation between the program coordinator and FMG nurses, who are considered essential partners for patients with CD. Although promising, these case management programs are often limited to the most serious cases due to capacity issues (50 persons per year at the CSSS de Chicoutimi).

Faced with the growing needs and primary care challenges posed by increasingly vulnerable patients, the *Agence de santé et de services sociaux* of the SLSJ region (regional health and social services agency) and the two partner CSSS proposed to implement similar and complementary primary care interventions to allow vulnerable patients with CD to benefit from case management by a nurse within their FMG. The expansion of case management within FMG will allow, together with the case management services already offered by the CSSS, a better response to the complex needs of vulnerable patients, as well as improved services integration. Case management will be performed in the primary care setting, the FMG, ensuring a better collaboration between case management nurse and family doctor [25]. The presence of a primary contact (FMG nurse), who is a generalist and accessible, will promote the coordination of patient care. As a result, the provision of case management can be adjusted in intensity and duration based on patient needs, ensuring continuity and long-term management.

The proposed intervention seeks to address many of the challenges posed by CD, based on scientific evidence. First, case management by primary care nurses has proven to be effective for various CD [25-27]. In fact, a key element of the effectiveness of an interdisciplinary approach to CD for vulnerable primary care patients is the use of a single caregiver (usually a nurse) to serve as the main contact and to coordinate interventions between health care professionals and the services provided [28]. However, most studies on this issue have been conducted in the context of a specific CD, which does not correspond to the reality of the clientele currently managed in primary care. Moreover, the implementation of strategies for interdisciplinary patient follow-up in the management of CD has to be evaluated in its context.

To date, strategies to support self-management remain poorly implemented in FMG. However, the positive effects of self-management support groups, such as the Stanford Chronic Disease Self-Management Program, are widely recognized [29,30]. These strategies are intended mostly for people who are in an early stage of their disease [31]. A recent study in primary care conducted in Ontario (Canada) showed a reduction in hospital length of stay and an increase in patient satisfaction with this type of self-management program [32]. Another study, in which vulnerable CD patients in primary care were evaluated after a six-week intervention to support self-management, showed positive effects with regard to patient self-management capacity, but there was no comparison with a control group [33]. Very few studies have examined the implementation mechanisms and the involvement of primary care professionals in such self-management support programs [34]. The introduction of a self-management group support program in FMG may inpsire more vulnerable people to participate and attain positive outcomes [4].

### Objectives

This project aims to document the implementation and effects, within four FMG of the SLSJ region (Quebec, Canada), of a pragmatic intervention involving case management by a nurse to promote interdisciplinary person-centred follow-up and group self-management support for frequent users of hospital services (emergency department visits and hospitalizations) with CD (diabetes, cardiovascular diseases, respiratory diseases, musculoskeletal diseases and/or chronic pain). The evaluation of the intervention has three objectives: (1) to analyze the implementation of the intervention within the existing structures of the four participating FMG in order to: (a) Explain how the various contexts have influenced the implementation of the intervention and the observed effects, and (b) Identify elements that can be assessed and applied in order to improve the intervention and to promote its implementation in other FMG; (2) to evaluate the proximal (self-efficacy, self-management practices, health habits, activation and psychological distress) and intermediate (empowerment, quality of life and health care use) effects of this intervention among patients; (3) Conduct an economic analysis of the cost-effectiveness and cost-benefit of the intervention.

### Conceptual framework

The theoretical framework of the intervention is based on two conceptual models. One supports the methodology of the clinical intervention, and the other supports the implementation process, change management, and knowledge transfer.

The first model is that of the UK National Health Service on innovation in health care and social services for people with CD [5]. This model incorporates the basic principles of the Chronic Care Model [35], while also drawing on lessons learned from US models, such as that of Kaiser Permanente, with regard to the intensity of care that is appropriate for the complex needs of patients [36], and of the Evercare model, with regard to the use of case management nurses in primary care [37]. The goal is to improve the health and quality of life of people with CD by providing personalized and ongoing support, based on the best evidence in the field. This model proposes the implementation of a case management system for patients with complex needs by making primary care a central part of the organization of services. To achieve this, the model suggests a structured approach that will allow for interaction between CSSS partners and community resources in order to provide integrated services. It also proposes the implementation of self-management support practices.

The second model the *Promoting Action on Research Implementation in Health Services* (PARiHS) [38,39]. According to this model, successful implementation depends on the nature and type of evidence from previous studies, the results of the proposed study, the context in which it is introduced, and how the process is facilitated. The value of the evidence depends not only on its scientific reliability, but also on the experience of the professionals and partners, as well as on patient preferences. The implementation of evidence into practice is achieved through a dialogue with knowledge users and must take their views into consideration. Some settings are more amenable to implementation than others, particularly where there are natural leaders. Finally, this model emphasizes the importance of appropriate facilitation, including various strategies for managing change and increasing the chances of a successful implementation.

## Methods/design

### Intervention

**Figure 1:**
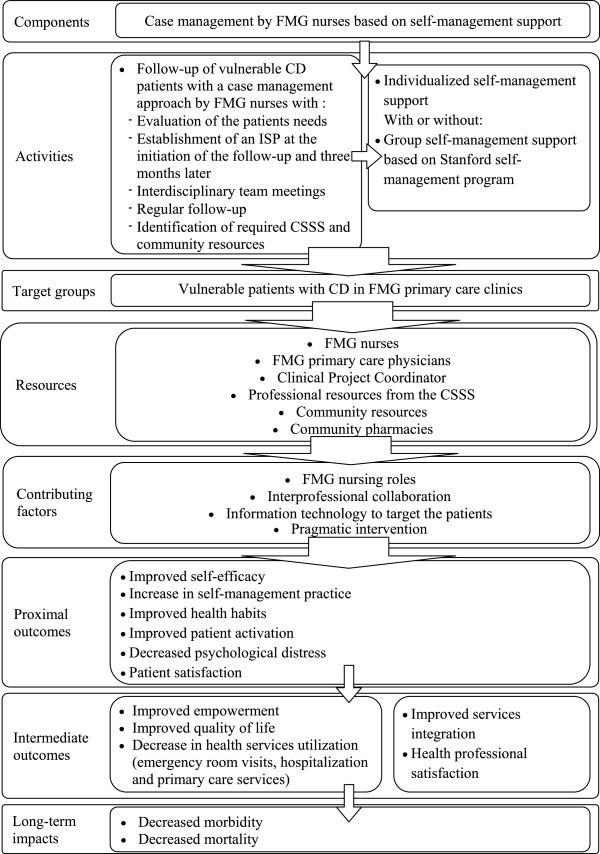
Intervention logic model.

Stemming from the theoretical framework described above, the components and activities of the intervention are shown in the logic model presented in Figure 1. The components of the intervention will include case management by FMG nurses and a self-management group support program as described below.

#### Case management by FMG nurses

The first component of the intervention is the follow-up offered under the case management process, which is seen as a collaborative, dynamic and systematic approach to ensure and coordinate care and services for a defined clientele based on interdisciplinary practice. Here the nurse evaluates, plans, implements, coordinates and prioritizes options and services according to patient health needs, in close collaboration with the involved partners [40]. The intervention focuses on four main components: (1) A thorough evaluation of patient needs and resources; (2) Establishment and maintenance of a patient-centred, individualized service plan (ISP); (3) Coordination of services among partners; and (4) Self-management support for patients and families [41].

The main duties and responsibilities of nurses in case management will be to: (1) Evaluate the patient’s situation and needs and involve the family, with the patient’s consent; (2) Identify which partners of the CSSS and of the community network to involve; (3) Jointly plan patient follow-up by establishing an ISP with the partners and with the active participation of the patients and family — minimum of two ISP per patient: one to initiate the intervention and the other approximately 3 months later; (4) Negotiate the services and defend the rights and interests of the individual; (5) Coordinate care and services; (6) Monitor the ISP application; (7) Educate and support the person. The ISP formulation stage will be oriented towards a self-management support approach that will emphasize the following: the patient’s potential; setting objectives according to his or her perspective; developing problem-solving skills; and using the patient’s usual support system [42]. Home visits by the nurse may be required, when justified — as in the case of reduced mobility or if a home assessment is needed — while avoiding overlap with home support services already offered.

The intervention will be implemented in each FMG by two nurses. Given the expertise of the nurses already working in the FMG and their established relationships with the medical teams and partners (e.g., community pharmacists), it is preferable that they implement the intervention rather than nurses hired for this project. This also promotes the sustainability of the intervention in the participating FMG. Nurses selected to conduct the intervention will receive five days of theory and practical training specific to case management and self-management support, provided by the Clinical Project Coordinator (described in following paragraph). Family doctors will be actively sought throughout the intervention to: identify participating patients, problems and intervention priorities, participate in ISP preparation meetings (in person or by having shared their perspective with the nurse prior to the meeting), and provide the medical components of ISP implementation. Partners include the CSSS professionals identified based on the needs of participants, such as psychosocial service providers (e.g. social worker) currently involved in a patient’s care, or additional professional resources specializing in CD management (nurse, nutritionist, etc.). The partners also include community organizations (e.g., home care services), patient associations and community pharmacists.

A Clinical Project Coordinator with experience in case management will be hired to coordinate and facilitate the implementation of the intervention (including change management). She will be responsible for: (1) setting up the intervention (training of case management nurses, development of materials and practice tools, recruitment and support of lay (peer) leaders for the self-management group support program, organization of support groups, scoping of organizations and community associations in the region and establishing the first contact to ensure their cooperation, etc.), including the communication plan to prepare participating FMG and all partners, in order to facilitate change management; and (2) ensuring the implementation and proper roll-out of the intervention (maintaining an appropriate standardized level of intervention and providing support for case management nurses, etc.). The Clinical Project Coordinator will be responsible for clinical governance in coordinating care within the network of each CSSS [43]. This clinical governance will allow the Clinical Project Coordinator to establish the required intervention treatment pathways, with the support of the two primary care managers involved, as needed.

#### Self-management group support program

The second component of the intervention consists of group meetings (10–12 participants) for self-management support in accordance with the Stanford program [3]. Developed by the Stanford Patient Education Centre (http://patienteducation.stanford.edu) to support people living with chronic conditions, the standardized curriculum, materials and program implementation, have made it the most accessible program for clinical and research applications [44]. An estimated 30% of participants enrolled in the project should agree to participate in this component, based on past primary care experiences described in the literature [32,33]. The self-management group support program rests on the premise that all patients with CD have similar concerns. They have the capacity to take responsibility for managing several aspects of their CD and will experience better results by building their sense of self-efficacy and developing the required skills. This model proposes a standardized six-week program with interactive weekly group meetings led by two lay leaders, who themselves have a CD. Twenty lay leaders (not participating in the intervention) will be recruited from the participating FMG practices and patient associations through a variety of media. Interviews will be conducted to ensure these volunteers are interested and able to provide this program. Two certified master trainers from *My Toolbox* program (http://mytoolbox.mcgill.ca) at McGill University will train lay leaders according to the Standard four-day training program. These lay leaders will conduct a complete simulation exercise with volunteer patients from their FMG to familiarize themselves with, and standardize, the process before conducting meetings with study participants. Two sessions of the self-management group support program will be implemented in each FMG. These meetings will take place at different times, depending on the availability of participants.

### Evaluation design

The evaluation of the intervention is based on a mixed design of complex health intervention evaluations [45]. It focuses on three combined strategies based on the targeted objectives and a pragmatic vision of the evaluation. These three strategies: the implementation analysis, the evaluation of the effects and the economic analysis will be described separately. The methodological decisions regarding patient selection, the implementation of the intervention and the intervention modalities were made in accordance with the established FMG practices to ensure the intervention meets their needs, and take into account the organization of services.

### Implementation analysis

This analysis will be based on two approaches: a realistic evaluation and a practical participatory approach. Consistent with the PARiHS conceptual framework, a realistic evaluation will help explain how different FMG, presenting a variety of practice environments from various perspectives, influence the implementation and the effects observed [46]. The realistic evaluation approach — described as pragmatic — recognizes that any outcome of an intervention results from the interaction between this intervention and its context [46]. It aims to highlight the underlying mechanisms and their performance under certain conditions. It recognizes that the outcomes are found not only in the patients but also in the stakeholders and organizations involved [47]. The three key questions of realistic evaluation are: (1) What has been implemented and why? (2) What were the obstacles/challenges and what were the facilitating conditions? (3) What explains the successes or failures (mechanisms)? The participatory approach will help identify the practical elements to be considered to improve the intervention and promote its implementation in other FMG [48]. The parameters to be evaluated have been identified to describe in detail the context (C), the intervention and its action mechanisms (I), and the effects of the intervention (E) on stakeholders and organizations, both qualitatively and quantitatively (Table 1).

**Table 1:** Parameters of the implementation evaluation

**Parameters evaluated**	**FMG stakeholders**	**Managers**	**Patients/ Family**	**Partners**	**Other sources**
**Pre-implementation phase**					
Description of practice settings (contextual factors) (C)	FG-II	II	FG	FG	
Description of the current processes, patient integration and satisfaction (C)	FG-II	II	FG	FG	
Issues related to the implementation (C)	FG-II	II	FG	FG	
**Implementation phase**					
Evolution of the processes and integration	FG	II			DR
Identification of problems and failures (C)	FG	II			
Fidelity of the intervention (I)					IFC
**Post-implementation phase**					
Opinion about the implementation process (C)	FG-II	II	FG	FG	
Identification of obstacles and facilitating elements (C)	FG-II	II	FG	FG	
Description of the impact on stakeholders/organizations (E)	FG-II	II	FG	FG	
Satisfaction with the intervention (E)	DG-II	II	DG	DG	

C: Context; I: Intervention; E: Effects.

FG: Focus group; II: Individual interview; DR: Documentation review; IFC: Intervention fidelity checklist.

#### Data collection strategies

A multiple-case study [49] will be performed with multiple sources of information. Such a strategy is particularly appropriate for analyzing the context and the mechanisms involved [50,51]. Each FMG will be considered a case with its own context, and will include its interactions with partners. This study will be conducted with a multifaceted approach before, during and after the intervention. As described in Table 1, four categories of key informants will be interviewed: (1) FMG stakeholders (doctors/nurses); (2) managers (FMG/CSSS); (3) participating patients and their families; (4) identified partners of case management (CSSS professionals, representatives of community organizations and community pharmacists), as well as volunteer leaders of self-management groups. Four strategies for collecting data will be used: (1) focus groups; (2) individual interviews; (3) review of documents produced during the implementation of the intervention, (4) an intervention fidelity checklist. Interview guides with open-ended questions that are adapted to each category of key informants will be developed and validated by implementation evaluation experts.

The pre-implementation phase will describe the context of the intervention regarding: (1) the characteristics of the environment (contextual factors); (2) the operation and integration of existing services, as well as participant satisfaction; (3) the issues related to the implementation as identified through group discussions. Focus groups will involve FMG doctors (n = 6, one group per FMG), patients and their families (n = 4 patients, one group per FMG; n = 4 family members, one group per FMG) and partners considered to be key participants (n = 4). Individual interviews will be conducted with FMG and CSSS managers (n = 6) and FMG nurses (n = 8).

The implementation phase will identify the changes in FMG processes (mechanisms) and the integration of services mid-way through the implementation in addition to learning about the obstacles and challenges encountered, as reported through focus groups with FMG stakeholders (n = 6) and obtained from individual interviews with FMG and CSSS managers (n = 6). Documentation from meetings of the advisory committee and from meetings between the Clinical Project Coordinator and nurses or managers, etc., will be obtained and analyzed in order to identify obstacles and challenges, and adjustments made along the way. An evaluation of the fidelity of the intervention will be conducted to document the degree to which the intervention was implemented; this will vary with the needs of participating patients [52]. After each encounter, nurses will conduct the evaluation for participating patients using a standardized checklist that includes the case management criteria and follow-up parameters, the details of ISP, the number of interdisciplinary meetings, the partners called upon and their involvement, and the number of self-management support meetings. These reports will help determine whether the interventions have conformed to the original study model.

The post-implementation phase will describe the implementation process, the obstacles and enabling factors, the effects of the intervention on stakeholders/organizations, and the satisfaction of key players. This will be accomplished through focus groups with FMG physicians (n = 6), patients and their families (n = 8) and partners considered key informants (n = 4), and individual interviews with FMG and CSSS managers (n = 6) and with FMG nurses (n = 8).

#### Data analysis plan

Data collected from the key stakeholders will be analyzed in three steps according to a qualitative content analysis procedure to identify emerging themes and trends: coding, sorting of documentation by content, and analysis. Driven by the data, inferences will be drawn and information units will be compared [53]. This content analysis will be performed using NVivo software (Version 9) for data from individual and group interviews. In addition to seeking to reveal the themes that are specific to each element of the implementation evaluation (context, intervention, mechanisms of action, impact), the analysis at this stage aims to: (1) explain how different contexts have influenced the implementation of the intervention and the observed effects; and (2) identify elements to be considered in order to improve the intervention and promote its implementation in the other FMG [50]. The scientific rigour of this approach will be assured by recognized qualitative research strategies [54]. Credibility (accuracy of the description of the phenomenon) will be assured by open-ended questions, allowing participants some latitude in what they want to reveal and by the triangulation of informants. Reliability (accuracy with which we account for various perceptions expressed by participants) will be assured by data coding by at least two members of the research team with expertise in different domains (triangulation of researchers). Validation (confirmation of the analysis and interpretation) will be assured through extensive documentation of the analysis process. Transferability (transfer of the outcomes to other settings or populations) will be assured by the detailed description of the context and participants.

### Evaluation of the effects

The evaluation of the effects on patients will be based on a pragmatic randomized experimental design with delayed intervention for the control group and measurements taken before and after the intervention (at six-month follow-up). This allows to evaluate interventions in actual clinical settings to maximize their generalizability [55]. Implementation analysis will document how different contexts influenced the implementation and the observed effects. The before/after experimental design is a robust method for observing the potential effects of the intervention [56]. The design also includes an assessment regarding use of services over a year in the experimental group.

#### Study population

Targeted patients will be those described in the objectives. The concept of vulnerability will be operationalized by considering both the frequency of the CSSS care use (emergency room visits and hospitalization) and patient vulnerability characteristics, based on the judgment of the primary care team in the FMG. This combined approach was proposed as a more favourable strategy for patient identification for this kind of intervention, when compared to using each one of these strategies in isolation [57]. Steps to target the most vulnerable participants will include: 1) Presentation of the intervention to FMG primary care physicians and nurses, 2) Delivery of a computerized list (obtained using MAGIC Chronique software by MédiaMed Technologies [58]) of the 300 most frequent users of CSSS services (emergency department visits and hospitalizations) having at least one of the targeted CD in each FMG. FMG professionals will then identify the 100 patients they believe will benefit most from the intervention described. The software used for patient identification will serve as a system to support decision-making for primary care teams who did not have access to this kind of information up till now.

After verifying patient eligibility and obtaining their consent, participants will be allocated to one of two groups (experimental or control) according to a three-stage randomization process [59]: (1) generation of the patient allocation sequence using a simple randomization procedure; (2) allocation blinding (sequentially numbered and sealed in opaque envelopes); (3) assignment of participants by opening the envelopes at the time of recruitment. The experimental group will consist of a sample of 50 patients in each of the four FMG (n = 200). These patients will receive the intervention for six months. Patients in the control group (n = 200) will receive the usual care for six months and then the same intervention as the treatment group for the following six months (waiting list control group) [60]. This is intended to avoid the problem of demoralization of the control group, in addition to ensuring fair treatment among participants in both groups.

### Measures

The variables defined within the logic model will be measured using instruments that are well known and have been validated in studies similar to the one proposed.

The proximal outcomes, evaluated at T0 (recruitment period), T1 (3 months) and T2 (6 months) for both groups (see Table 2), will be: (1) self-efficacy measured with the *Self-Efficacy for Managing Chronic Disease* scale [61]; (2) self-management practices, with the *Self-monitoring and Insight* sub-scale of the *health education impact questionnaire* (heiQ [62,63]; (3) health habits, using the questionnaire developed as part of the PRECISE study [64]; (4) patient activation, with the *Patient Activation Measure*[65]; and (5) psychological distress, with the *Psychological Distress Scale*[66].

**Table 2:** Proposed study plan

	**Visit 1**	**Visit 2**^**1**^	**Visit 3**	**Visit 4**	**T3**^**2**^
	**T0**		**T1**	**T2**	
Time from the start of the intervention	−2 weeks	0	3 months	6 months	12 months
Verification of eligibility	√				
Informed consent	√				
Covariables: Socioeconomic level, social isolation, literacy, mental health and multimorbidity	√				
Proximal outcomes: Personal self-efficacy, self-management practices, lifestyles, activation and psychological distress	√		√	√	
Intermediate outcomes: Empowerment, quality of life and use of services	√			√	√

^1^ Visit 2 will mark the beginning of the intervention. This will be two weeks after randomization for the experimental group and 6 months after randomization for the control group.

^2^ For the experimental group and use of services only.

Intermediate outcomes, evaluated at T0 and T2 (see Table 2), will include: (1) empowerment, measured with the heiQ scale [62]; (2) quality of life, measured with the SF-12v2 scale [67]; and (3) the use of health services, measured using clinical data from the CSSS (with Magic Chronique software by MediaMed) for hospitalizations, emergency room visits and CSSS services (e.g., psychosocial services or specialized services related to the specific CD), and electronic data on visits with physicians and nurses in the FMG.

A number of covariables will also be documented (T0) to describe participant characteristics: (1) socioeconomic status with family income and patient perceptions of his or her economic situation; (2) social isolation, measured with the social isolation subscale of the *Nottingham Health Profile*[68]; (3) literacy, measured with the *Newest Vital Sign*[69]; (4) mental health, measured with the *Hospital Anxiety and Depression Scale* (HADS) [70,71]; and (5) multimorbidity, measured with the *Disease Burden Morbidity Assessment* (DBMA) [72]. French versions are available for all validated instruments. When selecting data collection instruments, valid shorter versions were preferred to reduce completion time. Considering the circumstances of the vulnerable clientele, questionnaires will be self-administered in the presence of a research assistant who will provide the required assistance to the participant, ranging from minimal supervision to reading all the questions, if and when needed. If the patient cannot travel, the questionnaires can be administered by telephone or at the participant’s home.

The evaluation component will be conducted according to the study plan described in Table 2.

#### Sample size and statistical power

When analyzing quantitative data, we must take into account that part of the effect is potentially associated with each of the eight case management nurses. The intra-nurse or intracluster correlation (ρ) is unknown, however, and is specific to each variable. On the other hand, given that series of standardization measures will be implemented to minimize nurse-specific effects, we anticipate that the intracluster correlation will not be higher than 0.10. Sample size was therefore calculated using this maximal anticipated ρ-value. Results indicated that a standardized effect size (ES) of 0.5 (qualified as medium [73]) will be detectable with two groups of 200 participants (25 per nurse) for a two-tailed test with the α level set at 0.05 and a power of 80% [74]. An ES of at least this magnitude can be expected since it was observed in a previous study [75] and in unpublished data from the CSSS de Chicoutimi. An ES of at least 0.5 also represents the clinically significant effect needed to justify the pragmatic integration of the intervention into the services already provided. For example, in the case of patient empowerment, the ability to detect an ES of 0.5 translates into the capacity to detect any difference of at least half a point within different domains of the heiQ, since the overall standard deviation is about 1 point [76]. The detection of smaller differences is irrelevant since it is not clinically significant.

### Data analysis plan

All statistical analyses will be performed based on an intent-to-treat principle. We will first describe the characteristics of participants in each group, using means and standard deviations (continuous variables) or percentages (categorical variables). The groups will be compared at baseline (T0) using Student’s T test or the Chi-square test. Wherever possible, comparisons will be made between patients who agreed to participate in either phase of the study and those who refused, in order to document biases related to refusals. For outcome indicators at 3 months (self-efficacy, self-management practices, health habits, activation and psychological distress), groups will be compared at T1 using analysis of covariance adjusted for T0 scores. For indicators measured at six months (empowerment, quality of life and use of care), repeated measures analysis of variance will compare change over time in the two groups. In all cases, if the groups initially differ with regard to certain characteristics despite randomization, analysis will be adjusted to take into account the relevant variables. In addition, if a nurse effect is present (non-null intracluster correlation), despite efforts at standardization, multi-level analysis will be conducted to take this into account.

### Economic analysis

The economic analysis of the intervention will focus on a cost-effectiveness and a cost-benefit analysis. The implemented intervention and the usual care will be compared in each of these analyses.

The cost-effectiveness analysis (CEA) will compare the relative costs invested and effects of implemented intervention and usual care. On the other hand, the cost-benefit analysis will indicate the savings per dollar invested in the implemented intervention in terms of the benefit/cost ratio. Benefits will be assessed by assigning a monetary value to the effects. Because of the ethical and methodological problems in the assignment of a monetary value to quality of life, morbidity and mortality [77,78], the cost-benefit analysis will be based on a pragmatic approach; it will focus on the savings gained in relation to the reduction of health care use due to the implemented intervention.

### Stages of data acquisition and analysis

(1) **Cost analysis**. Cost analysis will be performed from an organizational perspective; that is, only costs related to the FMG and CSSS will be outlined. The costs of the intervention and of usual care will be identified. For the experimental group, the average nurse’s salary for the time devoted to intervention will be obtained (average cost per patient). The data on services obtained from the CSSS (emergency visits, hospitalizations, etc.) and from the FMG (excluding management of cases already included in the calculation) during the six-month follow-up will also be obtained and their monetary value will be estimated, using the average cost per patient for both groups. (2) **Measurement of effectiveness**. Data collected as part of the impact evaluation (intermediate outcomes) will be used to document the effects of the intervention on empowerment, quality of life, and use of services (emergency visits, hospitalizations, other CSSS services and FMG services). The effects will be measured in terms of the empowerment gained, quality of life gained, and, the reduction in use of services. (3) **Efficiency analysis**. Costs and effects will be compared, in order to identify whether the intervention or the usual care leads to better effectiveness at lower cost. Incremental cost-effectiveness ratios (ICER = Δ Effectiveness/Δ Costs) will be calculated. This component will address the following three questions: (1) How much does it cost to improve empowerment? (2) How much does it cost to improve quality of life? (3) How much does it cost to decrease use of CSSS or FMG services? (4) **Cost-benefit analysis**. A cost-benefit analysis will be performed if a reduction in the use of services is documented (see power calculation in “sample size and statistical power” section presented previously). The cost differential of the cost of services avoided, related to a reduction in the use of services for the number of patients surveyed (Δ benefits), will be compared to the cost differential of the intervention and the usual care (Δ costs) so as to assess whether the new intervention is cost-effective. We will calculate the gain per dollar invested in the intervention by estimating the Δ benefit/Δ cost ratio. If this ratio is greater than 1, a gain will be indicated; otherwise, a break-even point will be estimated by evaluating the minimum cost at which the intervention will become cost-effective. This analysis would also estimate the caseload for obtaining a break-even point and for identifying the profile of patients for whom the intervention was more cost-effective. (5) **Sensitivity analysis**. Since many uncertainties are generally present in the economic and effectiveness data, it will be important to complete the analysis by: (1) identifying and explaining the sources of uncertainty; and (2) performing sensitivity analyses by varying the value of specific parameters related to the sources of uncertainty, in order to assess the robustness of outcomes [77,79], and if necessary, to determine the break-even point of the intervention.

### Ethical considerations

This study was approved by the Research Ethics Board (REB) of the CSSS de Chicoutimi for both CSSS. Informed consent will be obtained from all participants. The six-month wait for the intervention for the control group has a negligible effect given the chronic nature of the conditions for which the intervention is proposed. Specific consent will be sought from each participating patient for access to their administrative health data and its use by the parties involved in the study. Confidentiality will be respected and data security ensured according to the rules in force within both CSSS and by the Research Ethics Board of the CSSS de Chicoutimi. Any publication resulting from this research will respect patient confidentiality.

## Discussion

The integration of case management by nurses and of self-management support groups into the FMG has the potential to impact patients positively, as outlined in the logic model. The long-term effects described therein cannot be measured due to the program's short timeframe, but may be confidently assumed from the evidence provided in the literature. FMG nurses will be able to continue to offer the intervention in their FMG after the implementation of the intervention. The caseload numbers that provide an optimal cost-benefit and a positive outcome profile for target patients will inform decision-makers and managers on the human and financial resources required to achieve optimal outcomes. In addition, decision-makers, managers and health care professionals will be aware of the factors to consider that favour the implementation of this intervention in FMG and other CSSS of the region and throughout Quebec.

### Strengths and limitations

The study design will not allow us to determine the individual effects of each component (case management and self-management group support) of the intervention. It was conceived in order to evaluate the addition of the self-management component for some patients who can benefit from it according to primary care practitioners’ perspective. Independent applications for funding are planned in order to evaluate the effects of each component in a near future. A contamination bias could occur between the case management nurses and the nurses involved with the patient control group. Several precautions will be taken to minimize this bias. First, no nurse will monitor both experimental group and control group. Discussions between nurses caring for the groups will be kept to a minimum with respect to the intervention and the new follow-up methods developed during the first six months of implementation. The implementation analysis will shed a qualitative light on this phenomenon. The presence of eight case management nurses raises the possibility of a “cluster” effect at the analysis level, which will be verified. However, this should be minimal since many precautions will be taken to reduce it: proper training of eight case management nurses to ensure standardization; the important role of the Clinical Project Coordinator in maintaining a comparable level of intervention among the eight nurses; regular discussions (every 1.5 months) among case management nurses. Repeat surveys induce a learning effect; however, the time-lapse between each survey questionnaire will be sufficient for the effect to be minimal. Regarding external validity, the pragmatic nature of the effects evaluation favours generalization. Analysis of the implementation process will identify the factors to be considered and the conditions to be put into place to support implementation of the intervention in other Quebec FMG.

## Abbreviations

CD: Chronic disease; CSSS: *Centre de santé et de services sociaux* / Health and social services centre; DBMA: Disease Burden Morbidity Assessment; DR: Documentation review; ES: Effect size; FMG: Family medicine group; FG: Focus group; FTE: Full-time equivalent; HADS: Hospital Anxiety and Depression Scale; heiQ: Health Education Impact Questionnaire; IFC: Intervention fidelity checklist; II: Individual interviews; ISP: Individualized service plan; PARiHS: Promoting Action on Research Implementation in Health Services; PRECISE: Program of research on the evolution of a cohort investigating health system effects; SLSJ: Saguenay-Lac-Saint-Jean

## Competing interests

This project is funded by the Pfizer-FRSQ-MSSS chronic disease fund. None of the funding agencies - Pfizer, *Fonds de recherche du Québec - Santé* (FRQ-S) or the *ministère de la santé et des services sociaux* (MSSS) - had any role in preparing, reviewing or approving the manuscript. They will not be involved in the collection, analysis or interpretation of the data.

## Authors’ contribution

MCC and CH contributed to the conception and design of the study and wrote the draft manuscript. MFD wrote the statistical methods and reviewed drafts of the manuscript. PR, MF and CL contributed to the description of vulnerable patients and measurement tools. They also reviewed and commented drafts of the manuscript. ET wrote the method on the economic evaluation and reviewed drafts of the manuscript. MF, EMC and MS revised the protocol to ensure the applicability of the intervention and that the methodology corresponds to the pragmatic perspective of the trial in the context of the primary care practices in which they are involved. All authors read and approved the final manuscript.

## Pre-publication history

The pre-publication history for this paper can be accessed here:

http://www.biomedcentral.com/1472-6963/13/49/prepub
